# Mindfulness Interfused with Humor: Insights From a Randomized Controlled Trial of a Humor-Enriched Mindfulness-Based Program

**DOI:** 10.1007/s12671-024-02491-7

**Published:** 2025-01-06

**Authors:** Christian T. Kastner

**Affiliations:** Applied Social and Health Psychology, Department of Psychology, University of Zurich, Binzmuehlestrasse 14/Box 14, 8050 Zurich, Switzerland

**Keywords:** Mindfulness-based program, Humor, Comic styles, Well-being, Life satisfaction, Worldview, Primal world beliefs

## Abstract

**Objectives:**

Both mindfulness and humor are inherently connected to well-being. Recent research found evidence for their combined effect in a joint training, the Humor-Enriched Mindfulness-Based Program (HEMBP). This study extends these findings by exploring (1) effects of Mindfulness-Based Stress Reduction (MBSR) on different forms of humor, (2) differential effects of the HEMBP on outcomes compared to MBSR, and (3) whether the HEMBP and MBSR may alter worldviews.

**Method:**

Ninety participants were randomly allocated to three conditions: the HEMBP, MBSR, and a wait-list control group. Participants’ mindfulness, psychological well-being, life satisfaction, perceived stress, comic styles, and primal world beliefs (primals) were assessed before and after the trainings, and at 1-, 3-, and 6-month follow-ups. Changes in outcome variables over time were modeled by applying linear mixed-effects models.

**Results:**

The HEMBP enhanced participants’ mindfulness, benevolent humor, psychological well-being, and life satisfaction compared to the wait-list control. Similarly, MBSR increased participants’ mindfulness and life satisfaction while reducing perceived stress and primal *good*, but no effects on humor were observed. Comparison between the two trainings revealed trends toward a greater increase in benevolent humor in the HEMBP group and a greater decrease in *good* in the MBSR group.

**Conclusion:**

The results largely replicate previous research on the efficacy of the HEMBP. Both programs demonstrated similar effects on outcomes, with only the HEMBP increasing benevolent humor and psychological well-being, while MBSR reduced stress. Further research is needed to investigate qualitative aspects of the integration of humor in MBPs and the long-term impact of MBPs on individuals’ worldviews.

**Preregistration:**

This study is not preregistered.

In recent years, the intersection of mindfulness and humor has emerged as a captivating area of exploration in psychological research (Geiger et al., 2019; Hofmann et al., 2020; Kastner, 2024; Özyeşil et al., 2013). Both are considered essential characteristics of humans and a crucial part of a mature and healthy personality (Kabat-Zinn, 1990; Martin, 2003). While individually recognized as important contributors to well-being (Goldberg et al., 2022; Martin & Ford, 2018), their combination in a joint training might yield synergetic effects due to shared mechanisms and attitudinal foundations (Kastner, 2024). This offers a promising avenue for facilitating continuous mindfulness practice and extending the reach of established mindfulness-based programs (MBPs; Loucks et al., 2022). The manuscript begins by presenting mindfulness, humor, and their intersection, introducing a mindful-humorous perspective, mindful interpersonal humor, and the mindful humor filter model. It then explores worldview as an integral part of the mindful humor filter model, examining its relationship with mindfulness, different forms of humor, and the possible malleability of worldviews. Next, the potential of MBPs—specifically the Humor-Enriched Mindfulness-Based Program (HEMBP)—to influence worldviews is discussed. Finally, the study’s objectives and hypotheses are outlined.

Mindfulness is defined as “a particular quality of intentional, continuous attention, characterized by an open, accepting, interested, and friendly attitude to present moment experiences” (Kastner, 2024, p. 2). It therefore always involves three essential, interconnected elements: intention, attention, and attitude (Shapiro et al., 2006). Humor is a broad concept that relates to everything funny, encompassing both benevolent and malicious forms of humor, while sense of humor refers to individual differences in humor perception, appreciation, and production (Martin & Ford, 2018). An important classification of the sense of humor is based on the comic styles, which categorize eight distinct elementary humor entities describing how individuals habitually engage in humor (Ruch et al., 2018a, b; Schmidt-Hidding, 1963). These styles are grouped into four lighter ones: benevolent humor, fun, wit, and nonsense, and four darker ones: irony, satire, sarcasm, and cynicism. Benevolent humor has the intention of arousing “sympathy and an understanding for the incongruities of life, the imperfections of the world, the shortcomings of fellow humans, and the own mishaps and blunders” (Ruch et al., 2018b, p. 3). This distinction is vital, as different forms of humor have varying relationships with mindfulness (Kastner, 2024) and well-being (Martin & Ford, 2018). For example, benevolent humor is positively associated with mindfulness (Kastner, 2024) and positive affect and life satisfaction (Ruch, Wagner, et al., 2018b), while sarcasm and cynicism correlate negatively with mindfulness (Kastner, 2024) and positively with negative affect (Ruch, Wagner, et al., 2018). In addition, the cross-cultural relevance of benevolent humor has already been demonstrated across 22 countries (Heintz et al., 2018).

The recently proposed mindful humor filter model (Fig. 1) delineates the mindfulness humor relationship, emphasizing overlaps in intention, attention, and attitude (Kastner, 2024). Although the attentional component of mindfulness might align with all forms of humor, only those styles or aspects of humor harmonizing with a non-harming intention and attitudinal foundations of mindfulness (e.g., openness, acceptance, interest, and friendliness) establish the basis for a mindful-humorous perspective and mindful interpersonal humor (Kastner, 2024). A mindful-humorous perspective is an internal outlook on life rooted in benevolent humor and, to a lesser extent, in neutral or benevolent aspects of fun, nonsense, and irony. It aims to cultivate well-being, understanding, and compassion for the world’s imperfections. Emerging from a profound experiential understanding of life’s ever-changing nature, this perspective blends the open, accepting attitude of mindfulness with a playful mindset, inviting a sense of lightheartedness into the present moment.

**Fig. 1:**
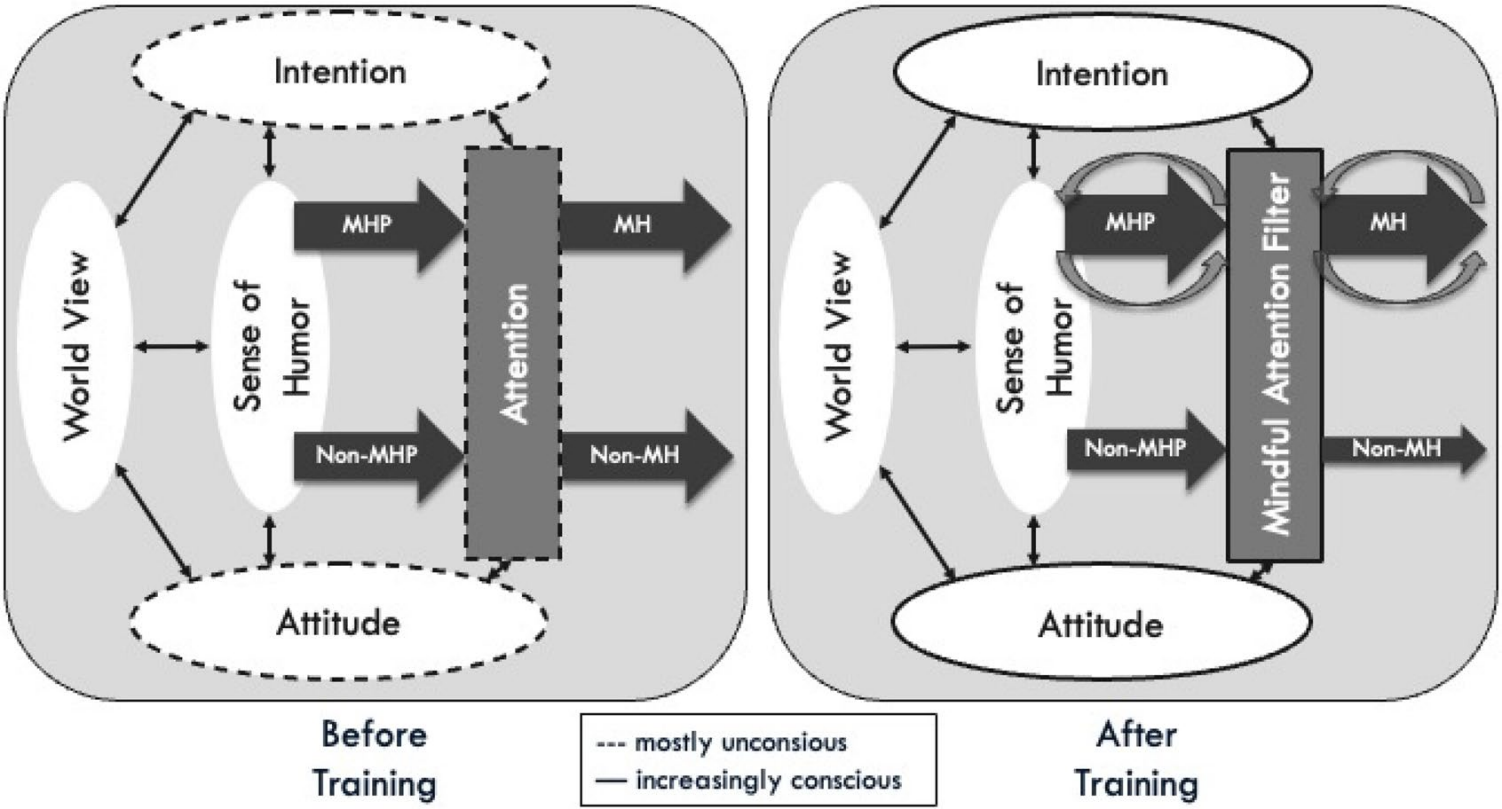
The Mindful Humor Filter Model (MHFM) before (left) and after (right) training. Mindfulness and humor are interconnected via intention, attention, and attitude, which are initially assumed to be mostly unconscious, thus depicted as dotted lines (left). With increasing levels of mindfulness (right), they become more and more conscious (solid lines), leading to an increase in the habitual occurrence of a mindful-humorous perspective (MHP) and mindful humor (MH) and a decrease in non-MHP and non-MH (e.g., sarcasm or cynicism). The decrease is assumed to be smaller for non-MHP, because the change in one’s habitual humor perspective is assumed to happen in the long-term, while the inhibition of non-MH may be easier achieved. MHP and MH both go along with a two-component positive reinforcement loop. The first is internal and refers to reinforcing shared mechanisms and attitudinal foundations through a MHP. The second is external and refers to MH fostering positive emotions and relationships. From “A lighthearted approach to mindfulness: development and evaluation of a humor-enriched mindfulness-based program in a randomized trial,” by C.T. Kastner, 2024, *Frontiers in Psychology*, *14, *1324329 (https://doi.org/10.3389/fpsyg.2023.1324329). CC BY-NC

A mindful-humorous perspective offers a complementary approach for reshaping one’s relationship with experiences. Within this paradigm, mindfulness and humor may synergistically support each other, drawing from potentially shared mechanisms and attitudinal foundations (Kastner, 2024). Both enable detachment from and reduced identification with thoughts, emotions, and sensations (Samson & Gross, 2012; Shapiro et al., 2006), leading to a shift in perspective known as reperceiving (Shapiro et al., 2006) or decentering (Segal et al., 2013). This shift, in turn, may facilitate the process of opening up to these experiences, accepting them, and exploring them with friendly curiosity, ultimately cultivating insights into the fundamental processes of life. A mindful-humorous perspective in meditation encourages individuals to approach hindrances like recurring thoughts with humor, fostering a lighthearted response. For example, when realizing excessive effort or attachment to outcomes, this perspective invites an internal smile, reflecting a deep awareness of the imperfections of life, easing the grip of striving or judgment, and promoting a positive and self-sustaining feedback loop for long-term mindfulness practice.

Such a perspective permeates daily life by discerning humor amid life’s incongruities, manifesting as mindful humor in interpersonal contexts (Kastner, 2024). Also grounded in benevolent humor, mindful humor incorporates more benevolent or neutral fun, and to a lesser extent, wit. Conversely, humor crossing into mockery, or ego-boosting, along with darker styles, is mostly mutually exclusive with mindfulness. However, darker styles emerging from a well-intended virtuous stance, such as gently correcting a behavior with irony, virtuous satire, or sporadic sarcasm or cynicism as sole or best coping mechanisms, may align with mindfulness. In interpersonal situations, a mindful case-by-case decision is necessary, considering others and the context, as every form of humor, even if well-intended, is susceptible to misunderstanding. Ideally, mindful humor has only positive effects for oneself and others fostering positive, mindful relationships. This, in turn, initiates another positive, self-sustaining feedback loop.

Worldviews may play an important role when gradually adopting a more mindful-humorous perspective on life. The mindful humor filter model (Fig. 1) incorporates the two feedback loops and proposes a dynamic relationship between mindfulness, sense of humor, and worldview via intention, attention, and attitude (Kastner, 2024). A worldview constitutes a collection of beliefs concerning physical and social reality, potentially exerting profound influences on cognition, emotion, motivation, and behavior (Koltko-Rivera, 2004). A crucial aspect of an individual’s worldview is its beliefs pertaining to the fundamental nature of the world on a most general level (i.e., basic attitudes), like the world is good, dangerous, or pleasurable, denoted as primal world beliefs (primals; Clifton et al., 2019). Primals exhibit robust correlations with numerous personality and well-being variables (Clifton et al., 2019; Stahlmann et al., 2020). The belief in the intrinsic goodness of the world (primary primal or general factor *good*) appears particularly significant, displaying strong associations with optimism, gratitude, curiosity, subjective well-being, flourishing, and key personality traits such as agreeableness and extraversion. *Good* embodies the belief that the world is inherently *safe*, *enticing*, and *alive* (secondary primals), reflecting an optimistic and benevolent outlook. As mindfulness is inherently based on attitudinal qualities like acceptance and compassion, with openhearted curiosity being integral to mindfulness practice (Kabat-Zinn, 2003), becoming more mindful may entail changes in one’s worldview so that it more strongly reflects the belief in a good, safe, enticing, and alive world.

The term “primal” suggests that these beliefs operate at a deep, foundational level, potentially influencing various aspects of an individual’s psychological functioning and, as an extension, health outcomes (Clifton et al., 2019). Thus, primals may also influence an individual’s sense of humor, and developing one’s sense of humor may co-occur with changes in primals. For example, a more pessimistic view of the world may manifest habitually through a more negative and distanced attitude, along with sarcastic thoughts, leading to implicit intentions for potentially hurtful or devaluating, sarcastic remarks. In contrast, a mindful-humorous perspective, reflected in an open, tolerant, and accepting attitude with an intention to arouse sympathy and understanding, may characterize the same situation, that could be expressed as mindful humor. One positive form of humor, humor as a character strength, correlated positively with the primary primal *good* and the secondary primals *safe*, *enticing*, and *alive* (Stahlmann & Ruch, 2023). Consequently, *good*, *safe*, *enticing*, and *alive* should be positively associated with transitioning to a more mindful-humorous perspective, resulting in positive correlations with mindfulness and benevolent humor and negative correlations with sarcasm and cynicism.

Primal world beliefs demonstrate considerable stability over time, with initial data indicating some independence from individual circumstances and life experiences (Clifton, 2020b), such as being privileged (Kerry et al., 2023) or even the COVID-19 pandemic (Ludwig et al., 2023). If so, consolidating specific primals holds potential for fostering long-term mental health. Consequently, a critical question arises: Is it even possible to modify primals, particularly at the highest hierarchical level, to enhance one’s sense of *good*? While existing evidence highlighting the significance of primals is largely correlational, and therefore, primals might merely be an outcome rather than a cause, a robust body of evidence substantiates the efficacy of Cognitive Behavioral Therapy and Beck’s premise (Beck, 1967), positing that cognition (i.e., beliefs about oneself, the future, and environment) shapes behavior (S. G. Hofmann et al., 2012). Furthermore, primals may primarily operate implicitly, rendering them more susceptible to change compared to, e.g., entrenched political beliefs (Clifton, 2020a). Clifton (2020a) outlined strategies for targeting primals in trainings, emphasizing the need for an experientially based approach supplemented by educational and motivational elements, as primals are less likely to undergo change through cognitive discussions alone. Instead of a top-down approach directly targeting primals, a bottom-up approach focusing on training specific behaviors or attitudes to bring about consequential changes in primals might be more effective.

A plausible assumption is that MBPs induce bottom-up changes in primals, owing to mindfulness’s transformative effects on cognition and behavior (Kabat-Zinn, 2003). MBPs primarily rely on first-person experience-mediated changes including educational and motivational elements (Crane et al., 2017), such as (re)connecting with personal strengths and aligning actions with one’s core values (Shapiro et al., 2006). As it is hypothesized that mindfulness is linked to sense of humor and worldview through bidirectional relationships involving intention, attention, and attitude (Fig. 1), mindfulness practice may impact not only one’s sense of humor but also one’s worldview in the long term (Kastner, 2024). Initially, an individual’s habitual use of humor, worldview, and underlying intention and attitudes are assumed to operate mostly implicitly. Then, with consistent mindfulness practice, a gradual shift toward a more habitual mindful-humorous, *good* perspective and mindful humor should be observable (Kastner, 2024).

Several factors may drive this transformation: (1) mindfulness practice strengthening intentional and attitudinal qualities underlying a mindful-humorous perspective and a *good* worldview, (2) increased mindfulness supporting the recognition of incongruities as a basis for humor, as well as of pleasant experiences (e.g., perceiving the good and beauty in the world), and (3) facilitating a present-centered space for assessing the appropriateness of humor, filtering out darker aspects of lighter forms of humor and inhibiting, reducing, or transforming destructive forms, such as sarcasms or cynicism. Finally, mindful awareness may enhance recognition of automatic and implicit thought patterns without becoming entangled, facilitating a more balanced and realistic perception of the world. For example, the efficacy of MBPs in reducing negative thought patterns associated with anxiety and depression, such as rumination and worry, is well-documented (Gu et al., 2015), probably altering pessimistic worldviews (e.g., decreased negative cognitive biases; Ford et al., 2023).

The HEMBP was developed based on the mindful humor filter model, explicitly aiming at fostering a mindful-humorous perspective and mindful humor (Kastner, 2024). Preliminary results have shown that the HEMBP was effective in fostering psychological well-being and reducing stress (Kastner, 2024). Further, the validity of the HEMBP was demonstrated by finding evidence for its positive effects on mindfulness as well as distinct effects on comic styles. In line with the mindful humor filter model, participation in the HEMBP was associated with an increase in benevolent humor, and a decrease in cynicism, sarcasm, and the fear of being laughed at (gelotophobia). Although this first randomized controlled trial showed promising results regarding the efficacy of the HEMBP and provided initial evidence for the validity of the mindful humor filter model, the follow-up period of 1 month was relatively short. Further, Kastner (2024) only included a wait-list group as a control condition without comparing the effects to a standard mindfulness training such as MBSR, and it lacked a measure of worldviews.

This study seeks to extend the findings of Kastner (2024) through a randomized controlled trial with a prolonged follow-up and by introducing an active control group. One objective is to assess the validity and efficacy of the HEMBP compared to mindfulness-based stress reduction (MBSR; Kabat-Zinn, 1990). MBSR is commonly acknowledged as the “gold standard” among MBPs, supported by a substantial body of evidence demonstrating its positive effects on well-being and stress, across clinical and non-clinical populations (Goldberg et al., 2022). Moreover, this design enables the exploration of potential differential effects of the HEMBP on outcomes compared to MBSR, which might serve as indicators for synergies resulting from the combination of mindfulness and humor. This study also allows testing the theoretically assumed differential effects of a mindfulness-only training (MBSR) on benevolent humor, sarcasm, and cynicism, as outlined in the mindful humor filter model. Lastly, the study explores whether participation in MBPs may even have the capacity to influence one’s worldviews. It is hypothesized that participation in the HEMBP and MBSR, respectively, leads over time to a greater increase in mindfulness, benevolent humor, psychological well-being, and life satisfaction, and to a greater reduction in sarcasm, cynicism, and stress compared to a wait-list control group. Further, it is expected that the effects for the HEMBP exceed those for MBSR regarding benevolent humor, sarcasm, and cynicism. Lastly, it is hypothesized that participation in the HEMBP and MBSR, respectively, is associated with an increase in the primary primal *good*, while effects on the secondary primals are investigated exploratively. Although one tertiary primal explicitly pertains to laughter and humor, termed *funny*, it does not allow to distinguish between different humor qualities (e.g., “Laughing a ton makes sense because life is hilarious and humor is everywhere.”), thus, it is investigated exploratively.

## Method

### Participants

Eligible participants for this study were adults aged 18 years and older, with adequate knowledge of German, no prior experience in mindfulness meditation, and not receiving psychotherapeutic treatment at the time. The final sample consisted of 90 participants (28.89% male, 70.00% female, 1.11% not specified) between 20 and 62 years of age (*M* = 38.29, *SD* = 12.41) who were randomly assigned to one of the three conditions (each *n* = 30): HEMBP, MBSR, or the wait-list control group (WL). The demographic data of participants is shown in Table 1.

**Table 1:** Participant demographics and results of between-group comparisons at pre-test and by completion status

Variable	Pre-test					Completion	
	HEMBP (*n* = 29)	MBSR (*n* = 30)	WL (*n* = 30)	*χ*^2^ (*df*) (*N* = 89)	*p*	*χ*^2^ (*df*) (*N* = 89)	*p*
Gender, *n* (%)				2.32 (1)	0.68	0.25 (2)	0.88
Male	7 (24.14%)	9 (30.00%)	9 (30.00%)				
Female	21 (72.41%)	21 (70.00%)	21 (70.00%)				
Not specified	1 (3.45%)	0 (0.00%)	0 (0.00%)				
Highest level of education, *n* (%)				6.07 (8)	0.64	4.82 (4)	0.31
Apprenticeship	2 (6.90%)	6 (20.00%)	3 (10.00%)				
Baccalaureate school	4 (13.79%)	2 (6.67%)	4 (13.33%)				
Bachelor’s degree	10 (34.48%)	6 (20.00%)	6 (20.00%)				
Master’s degree	10 (34.48%)	11 (36.67%)	14 (46.67%)				
Doctorate	3 (10.34%)	5 (16.67%)	3 (10.00%)				
Nationality, *n* (%)				8.23 (6)	0.22	2.72 (3)	0.44
Swiss	17 (58.62%)	27 (90.00%)	22 (73.33%)				
German	5 (17.24%)	2 (6.67%)	4 (13.33%)				
Austrian	2 (6.90%)	0 (0.00%)	1 (3.33%)				
Other	5 (17.24%)	1 (3.33%)	3 (10.00%)				
Marital status, *n* (%)				5.66 (6)	0.46	1.32 (3)	0.72
Single	14 (48.28%)	8 (26.67%)	10 (33.33%)				
Partnership	8 (27.58%)	7 (23.33%)	8 (26.67%)				
Married or registered partnership	7 (24.14%)	13 (43.33%)	10 (33.33%)				
Divorced	0 (0.00%)	2 (6.67%)	2 (6.67%)				
Employment status^a^, *n* (%)							
Working full-time	10 (34.48%)	12 (40.00%)	13 (43.33%)	0.49 (2)	0.78	0.03 (1)	0.86
Working part-time	12 (41.38%)	18 (60.00%)	14 (46.67%)	2.18 (2)	0.34	1.68 (1)	0.20
Studying full-time	8 (27.59%)	0 (0.00%)	3 (10.00%)	10.59 (2)	0.01	2.42 (1)	0.12
Studying part-time	3 (10.34%)	6 (20.00%)	2 (6.67%)	2.62 (2)	0.27	0.01 (1)	0.93
Housekeeping	1 (3.45%)	1 (3.33%)	2 (6.67%)	0.50 (2)	0.78	2.59 (1)	0.11
Retirement	0 (0.00%)	0 (0.00%)	0 (0.00%)				
Unemployment	1 (3.45%)	0 (0.00%)	1 (3.33%)	1.04 (2)	0.59	1.26 (1)	0.26
Income (CHF), *n* (%)				16.39 (8)	0.04	1.27 (4)	0.87
≤ 3000	11 (37.93%)	2 (6.67%)	9 (30.00%)				
3001–6000	7 (24.14%)	14 (46.67%)	10 (33.33%)				
6001–10,000	8 (27.59%)	8 (26.67%)	8 (26.67%)				
> 10,000	2 (6.90%)	1 (3.33%)	3 (10.00%)				
Not specified	1 (3.45%)	5 (16.67%)	0 (0.00%)				
				*F* (2,89)		*t* (87)	
Age, *M* (*SD*)	34.69 (11.38)	42.33 (13.18)	37.73 (11.99)	2.93	0.06	0.11	0.92

*HEMBP*, humor-enriched mindfulness-based program; *MBSR*, mindfulness-based stress reduction; *WL*, wait-list control group; *Completion*, comparison between completers (*n* = 71) and non-completers (*n* = 18). ^a^For employment status, multiple responses were possible. Therefore, the *n* and percentage in each cell represent the individuals that selected the respective option out of 29 each (HEMBP) or out of 30 each (MBSR, WL)

**Fig. 2:**
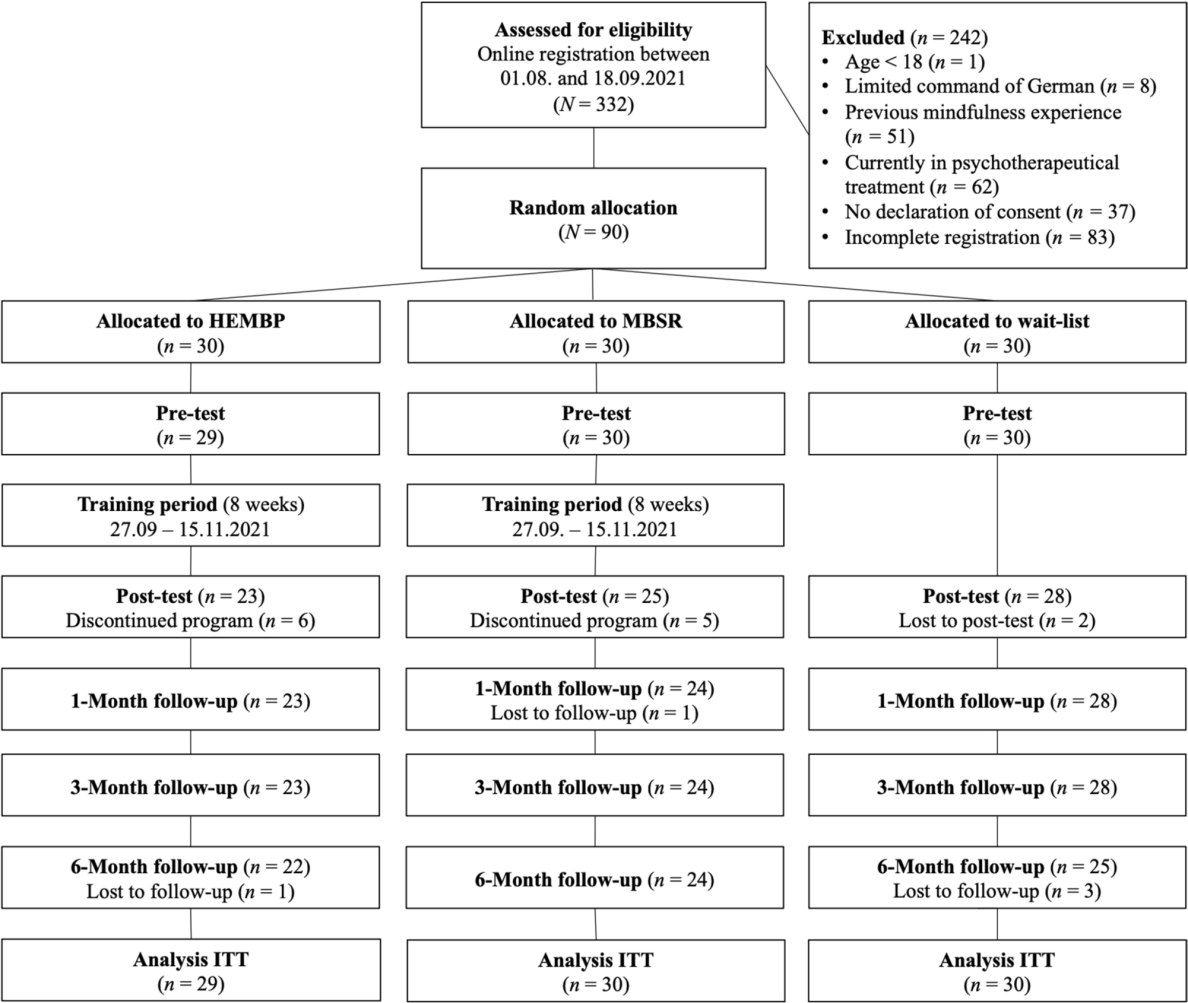
Participants flow chart in accordance with CONSORT criteria. HEMBP, humor-enriched mindfulness-based program; MBSR, mindfulness-based stress reduction; ITT, intention-to-treat

Most participants were Swiss (73.33%), German (13.33%), or Austrian (3.33%). More than two-thirds of participants had a bachelor’s degree or higher (76.67%). The majority of participants was employed either full-time (*n* = 36) or part-time (*n* = 44) or was studying full-time (*n* = 22) or part-time (*n* = 11). Throughout the study, 79.78% of the sample was retained, as shown in the participants flow chart (Fig. 2): 89 participants completed the pre-test, 76 the post-test, 75 the 1-month follow-up, 75 the 3-month follow-up, and 71 the 6-month follow-up. The reasons for training discontinuation were time constraints (*n* = 6), family bereavement (*n* = 2), and a decline in physical health (*n* = 2), evenly distributed among the HEMBP and MBSR, as well as personal reasons (*n* = 1; HEMBP only).

### Procedure

Participants were recruited from August 1st to September 18th, 2021, via a radio interview, a project website, mailings to companies, flyers, and social networks, all of which included a link to the online registration platform. Participants who met eligibility requirements received comprehensive information regarding the study. Subsequently, written consent was obtained from them, and basic demographic data was collected. To provide an incentive, personalized feedback was offered based on the responses provided in the self-reports. Participants were required to make a payment of CHF 100 to enhance motivation and minimize the likelihood of participant attrition. Participants were matched based on their gender and randomly assigned to one of the three conditions. The randomization process was conducted using a computer-generated list that remained concealed from the primary investigator until all participants were assigned. Throughout the study, all participants were requested to complete the same set of self-reports online on five occasions: 1 week prior to the start of the trainings and 1 week, 1 month, 3 months, and 6 months after completion of the trainings.

The training sessions were conducted as face-to-face group programs. The quality of program delivery was ensured by dividing participants into sub-groups, each comprising 15 individuals. The trainings took place in classrooms at the University of Zurich, between September 27th and November 15th, 2021. Participants gathered on eight consecutive Mondays, with each session lasting 2 hr and 15 min, and for a half-day retreat (6.50 hr). Following the completion of data collection in June 2022, the individuals in the wait-list control group were also provided with the HEMBP. The MBSR group was led by an MBSR teacher with 10 years of teaching experience. The HEMBP group was led by an MBSR/HEMBP teacher with 5 years of teaching experience. Both teachers were certified MBSR instructors recognized by the Swiss Mindfulness Association and followed the respective standard curricula for conducting the training sessions. However, sessions were shortened to 2 hr and 15 min to allow the two sub-groups of each program to occur consecutively on the same evening. Between-class assignments (≥ 30–40 min/day, 6 days/week) entailed engaging in formal and informal mindfulness practices, exercises, and utilizing calendars. There was no orientation session for participants prior to the study.

The HEMBP was developed by restructuring MBSR to incrementally integrate playful and humorous elements (Kastner, 2024). While the HEMBP was designed as a standalone MBP, it is similar in many ways to the structure and teaching process of MBSR. The basic structures of the weekly sessions, the day-long session, and the first and last classes are similar to MBSR. Likewise, the introduction of core meditation practices follows a similar order and class arrangement. However, a tree meditation was introduced in the first class and loving-kindness meditation in the second. The guided formal meditation recordings were abbreviated to 20 min. In the HEMBP, Class 3 aligns with Class 2 in MBSR, while Class 7 covers unpleasant experiences and stress as a combination of Classes 4 and 5 of MBSR. Notable modifications were implemented in Classes 2, 4, 5, and 6 of the HEMBP (see course overview in Kastner, 2024).

### Measures

#### Comic Style Markers (CSM; Ruch et al., 2018a, b)

The CSM questionnaire consists of 48 items measuring eight comic styles: fun, benevolent humor, nonsense, wit, irony, satire, sarcasm, and cynicism. Each scale comprises six items, utilizing a 7-point response format from 1 (*strongly disagree*) to 7 (*strongly agree*). In the current study, the internal consistency across measurement occasions ranged from *α* = 0.64 (benevolent humor) to 0.93 (nonsense) and from *ω* = 0.76 (benevolent humor) to 0.96 (nonsense).

#### Comprehensive Inventory of Mindfulness Experiences (CHIME; Bergomi et al., 2014)

The CHIME offers a multi-dimensional assessment of mindfulness on eight subscales: inner awareness, outer awareness, acting with awareness, openness to experiences, accepting and non-judgmental orientation, decentering and non-reactivity, insightful understanding, and relativity of thoughts. Participants provide answers on a 6-point scale from 1 (*almost never*) to 6 (*almost always*). In this study, internal consistency across measurement occasions ranged from *α* = 0.90 to 0.94 and from *ω* = 0.92 to 0.96.

#### Comprehensive Inventory of Thriving (CIT; Su et al., 2014)

The CIT assesses subjective as well as psychological well-being (positive functioning) across 18 subscales that cover seven well-being dimensions: relationships, engagement, mastery, autonomy, meaning, optimism, and subjective well-being. It comprises 54 items typically rated on a 5-point Likert scale; however, for the purpose of the current study, the response format has been extended to a 7-point scale, ranging from 1 (*strongly disagree*) to 7 (*strongly agree*) to accommodate for potential ceiling effects. In this study, the German version (Hausler et al., 2017) was employed with internal consistency across measurement occasions ranging from *α* = 0.95 to 0.97 and from *ω* = 0.97 to 0.98.

#### Perceived Stress Scale-10 (PSS-10; Cohen et al., 1983)

The PSS measures perceived stress over the preceding month. The scale consists of 10 items with a 5-point Likert scale from 1 (*never*) to 5 (*very often*). The 10-item German version of the PSS was used (Klein et al., 2016), with internal consistency ranging from *α* = 0.81 to 0.87 and from *ω* = 0.87 to 0.92 across measurement occasions.

#### Satisfaction with Life Scale (SWLS; Diener et al., 1985)

The SWLS is a 5-item questionnaire designed to measure an individual’s general satisfaction with life. It uses a 7-point scale ranging from 1 (*strongly agree*) to 7 (*strongly disagree*). In this study, the German version (Ruch et al., 2010) of the scale was used. Internal consistency was between *α* = 0.85 and 0.90 and between *ω* = 0.89 and 0.93 across measurement occasions.

#### Primal World Beliefs Inventory (PI; Clifton et al., 2019)

The PI measures 26 primal world beliefs, referred to as primals, concerning the general character of the world with 99 items on a 6-point Likert scale from 1 (*strongly disagree*) to 6 (*strongly agree*). In the present study, the 66-item German version (Stahlmann et al., 2020) was administered to measure the primary primal *good*, the six secondary primals of the German version (*safe*, *enticing*, *alive*, *fluid*, *empowering*, *communal*), and the tertiary primal *funny*. The internal consistencies across measurement occasions ranged from *α* = 0.55 (*funny*) to 0.95 (*good*) and *ω* = 0.63 (*funny*) to 0.96 (*good*).

#### Hospital Anxiety Depression Scale (HADS; Zigmond & Snaith, 1983)

The HADS comprises 14 items assessing symptoms of anxiety and depression on two separate dimensions with seven items each, ranging from 0 (e.g., *not at all*) to 3 (e.g., *most of the time*). The 14-item German version (Herrmann-Lingen et al., 2011) was used in this study. Internal consistency at pre-test was *α* = 0.79 and *ω* = 0.85 for anxiety and *α* = 0.79 and *ω* = 0.86 for depression. The HADS was employed for a manipulation check only, that is, to investigate whether randomization created comparable groups at pre-test.

To enhance the ability to detect changes, the timeframe referred to by the items in the following questionnaires was restricted to the past 2 weeks: CSM, CIT, PSS, SWLS, and Primals.

### Data Analyses

The sample size was determined using a priori power analyses conducted with G*Power 3.1 (Erdfelder et al., 2009). Sedlmeier et al. (2017) estimated a global effect size of $\overline{r }=0.$ 27 for meditation studies involving healthy participants. Assuming *α* = 0.05 and power = 0.95, and anticipating a correlation of 0.40 among repeated measures, the sample size for a repeated measures design testing a within-between interaction was determined to be at least *n* = 42.

Potential group differences at pre-test for demographic and outcome variables were examined with one-way analysis of variance (ANOVA) for continuous variables and chi-square tests for categorical variables. To explore potential differences between completers and dropouts, a series of *t* tests (for continuous variables) and chi-square tests (for categorical variables) was employed.

The repeated measures taken 1 week prior to, and 1 week, 1 month, 3 months, and 6 months after the program (level 1) are nested within participants (level 2). Given the hierarchical data structure, a series of linear mixed-effects models was employed. The “lme” function from the R package “nlme” (Pinheiro et al., 2021) was used for the analyses in R (R Core Team, 2021), which estimated the models using restricted maximum likelihood. These models can estimate model coefficients even in the presence of missing data in the outcome variables. Therefore, for intention-to-treat analyses following CONSORT guidelines (Schulz et al., 2010), the application of multiple imputation is not necessary (Twisk et al., 2013).

To investigate how to best incorporate time, model comparisons were undertaken, considering three model types with diverging flexibility regarding the shape of change. Firstly, the least flexible model considered was a simple growth model, which assumes linear change across all measurement occasions, with time coded as 0 2 3 5 8. Secondly, as a semi-flexible variant, a piecewise-growth model was considered that divided the time predictor into two distinct phases. Phase 1 captures linear change across the training period from pre- to post-test, coded as 0 2 2 2 2, and phase 2 captures linear change or stability during the 6-month follow-up period, coded as 0 0 1 3 6. Lastly, the most flexible variant included time as a factor, which results in separate estimates of change between the first (reference category) and each later measurement occasion. Unlike the previous (piecewise) linear models, this model makes no assumption regarding the overall shape of change. All models included a random intercept, but fixed slopes for the time predictors. Otherwise, it would have been necessary in the first model when adding time as a factor to fix the residual variance at level 1 to a value close to 0 (Lischetzke et al., 2015). This would have rendered model comparison based on the Akaike Information Criterion (AIC) unfeasible. Comparisons between the three models using the AIC indicated that, for nearly all outcomes, the growth model consistently exhibited the lowest AIC scores. However, in the case of mindfulness, the piecewise-growth model yielded a lower AIC score. Consequently, results for mindfulness are reported based on the piecewise-growth model, while results for all other outcomes are presented using the linear growth models.

Training effects were assessed by analyzing the cross-level interactions between time and condition, reflecting differences in the rate of change over time between the training groups and the wait-list control. Regarding mindfulness, the piecewise-growth model included two interaction terms, one for each time phase. In all models, time was coded as the number of months from the start of the trainings, ranging from 0 at pre-test to 8 at the last follow-up in the growth models and from 0 to 6 in the piecewise-growth model, respectively. Consequently, the interaction estimates can be interpreted as the change in the outcome variables for a one-unit change in time (i.e., 1 month). In order to assess differences between the HEMBP and MBSR, the reference category was switched between the wait-list control and the HEMBP.

Correlations between average practice time (during the training period and the follow-up period, respectively) and between change scores of the outcome variables (pre- to post-test, and post-test to the last follow-up) were investigated exploratively, separately for the two trainings. Positive values indicate that longer formal mindfulness practice is positively related to larger changes in outcome variables between pre- and post-test (training period), and post-test to last follow-up period (follow-up period). Group differences in daily formal practice time were assessed with separate *t* tests.

## Results

No significant between-group differences at pre-test were identified for the following variables: age, gender, nationality, civil status, education, and employment status (except for studying full-time). Significant between-group differences were found for income and studying full-time. No significant differences were observed among the three groups at pre-test regarding mean scores of mindfulness, fun, benevolent humor, nonsense, wit, irony, satire, sarcasm, cynicism, psychological well-being, life satisfaction, perceived stress, primal world beliefs, as well as symptoms of anxiety and depression. Test statistics for group comparisons at pre-test are presented in Table 1 (demographics) and Table 2 (outcome variables), while descriptive data (means and standard deviations) for all outcome variables can be found in Table 3. Additionally, Table 4 displays the correlation matrix for all outcome variables at pre-test, providing a more comprehensive understanding of the correlations between them.

**Table 2:** Results of between-group comparisons for outcome variables at pre-test and by completion status

Outcome	Pre-test		Completion	
	*F* (2,86)	*p*	*t* (87)	*p*
Mindfulness	0.55	0.58	1.71	0.09
Fun	< 0.01	0.99	− 0.54	0.59
Humor	1.47	0.24	1.54	0.13
Nonsense	1.25	0.29	0.19	0.85
Wit	0.21	0.81	0.26	0.80
Irony	0.62	0.54	0.74	0.46
Satire	1.35	0.26	− 0.09	0.93
Sarcasm	0.09	0.91	− 1.82	0.07
Cynicism	1.25	0.29	0.15	0.90
PWB	0.68	0.51	0.93	0.35
Life Satisfaction	0.60	0.55	2.17	0.03
Stress	0.59	0.56	− 0.74	0.46
Good	0.17	0.84	0.51	0.61
Safe	0.42	0.66	1.25	0.22
Enticing	0.59	0.56	− 0.36	0.72
Alive	0.52	0.60	0.06	0.95
Fluid	0.34	0.71	− 1.25	0.21
Empowering	0.65	0.53	− 0.63	0.53
Communal	0.26	0.77	0.38	0.71
Funny	1.44	0.24	1.14	0.26
Anxiety	0.06	0.94	− 0.71	0.48
Depression	0.10	0.91	− 1.22	0.23

*PWB*, psychological well-being; *Completion*, comparison between completers (*n* = 71) and non-completers (*n* = 18)

**Table 3:** Descriptive data of outcome variables across five measurement occasions for the three conditions

Outcome	Pre-test			Post-test			1-month FU			3-month FU			6-month FU		
	*n*	*M*	*SD*	*n*	*M*	*SD*	*n*	*M*	*SD*	*n*	*M*	*SD*	*n*	*M*	*SD*
Mindfulness															
WL	30	3.54	0.60	28	3.69	0.59	28	3.58	0.62	28	3.63	0.58	26	3.65	0.67
HEMBP	29	3.69	0.48	23	4.04	0.52	23	4.04	0.56	23	4.02	0.56	22	4.02	0.59
MBSR	30	3.65	0.52	25	4.06	0.39	24	4.06	0.49	24	4.14	0.49	24	3.99	0.52
Fun															
WL	30	4.22	1.11	28	4.30	1.01	28	4.49	1.01	28	4.44	0.97	26	4.32	1.00
HEMBP	29	4.24	0.82	23	4.33	0.94	23	4.41	0.82	23	4.28	0.97	22	4.23	1.07
MBSR	30	4.23	1.13	25	4.27	1.10	23	4.15	1.16	24	4.13	1.13	24	4.26	1.10
Humor															
WL	30	4.64	1.06	28	4.76	0.96	28	4.71	1.04	28	4.65	0.95	26	4.53	1.06
HEMBP	29	4.74	0.74	23	4.90	0.54	23	4.81	0.67	23	5.01	0.61	22	5.02	0.73
MBSR	30	5.01	0.74	25	5.13	0.53	23	5.03	0.73	24	5.01	0.68	24	5.08	0.84
Nonsense															
WL	30	4.62	1.05	28	4.63	1.04	28	4.46	1.33	28	4.44	1.22	26	4.36	1.24
HEMBP	29	4.87	1.02	23	5.04	1.10	23	4.88	1.32	23	4.95	1.04	22	4.89	1.17
MBSR	30	4.42	1.25	25	4.59	1.34	23	4.28	1.41	24	4.22	1.82	24	4.45	1.52
Wit															
WL	30	4.28	1.11	28	4.30	1.11	28	4.47	1.15	28	4.33	1.14	26	4.24	1.04
HEMBP	29	4.41	1.09	23	4.70	1.07	23	4.68	1.08	23	4.70	1.07	22	4.73	1.06
MBSR	30	4.23	1.11	25	4.25	1.08	23	4.37	1.19	24	4.26	1.02	24	4.48	0.99
Irony															
WL	30	3.77	0.96	28	3.99	0.86	28	4.14	0.90	28	3.93	0.78	26	3.96	1.03
HEMBP	29	3.98	1.22	23	4.01	1.24	23	3.88	1.22	23	3.97	1.10	22	3.78	1.17
MBSR	30	4.06	0.98	25	3.99	0.96	23	3.96	0.90	24	3.76	1.05	24	3.88	1.06
Satire															
WL	30	3.62	0.99	28	3.76	1.19	28	3.82	1.12	28	3.71	1.10	26	3.61	1.30
HEMBP	29	3.93	1.09	23	3.98	1.27	23	3.75	1.21	23	4.01	0.97	22	4.02	1.33
MBSR	30	4.01	0.80	25	3.92	0.98	23	3.97	1.08	24	3.97	1.10	24	4.20	0.93
Sarcasm															
WL	30	3.37	1.11	28	3.31	1.18	28	3.25	1.04	28	3.24	1.16	26	3.28	1.17
HEMBP	29	3.31	1.25	23	3.43	1.40	23	3.50	1.46	23	3.44	1.18	22	3.36	1.33
MBSR	30	3.45	1.38	25	3.17	1.21	23	3.20	1.09	24	3.35	1.31	24	3.41	1.37
Cynicism															
WL	30	3.26	1.01	28	3.46	1.19	28	3.44	1.11	28	3.46	1.10	26	3.27	1.10
HEMBP	29	3.13	1.11	23	3.15	1.18	23	3.28	1.40	23	3.38	1.07	22	3.31	1.13
MBSR	30	3.57	1.16	25	3.29	0.88	23	3.48	1.13	24	3.53	1.27	24	3.62	1.26
PWB															
WL	30	5.17	0.72	28	5.16	0.80	28	5.22	0.94	28	5.12	0.66	26	5.07	0.90
HEMBP	29	5.05	0.75	23	5.21	0.88	23	5.19	0.92	23	5.30	0.86	22	5.31	0.86
MBSR	30	5.26	0.61	25	5.39	0.47	24	5.38	0.56	24	5.49	0.60	24	5.46	0.63
Life satisfaction															
WL	30	4.75	1.02	28	4.76	1.14	28	4.91	1.30	28	4.65	1.16	26	4.66	1.22
HEMBP	29	4.54	1.25	23	4.71	1.21	23	4.87	1.27	23	4.83	1.09	22	4.94	1.09
MBSR	30	4.85	1.02	25	4.98	0.77	24	5.01	0.89	24	5.18	1.06	24	5.24	0.90
Stress															
WL	30	2.88	0.63	28	2.75	0.52	28	2.89	0.65	28	2.94	0.51	26	2.95	0.65
HEMBP	29	2.92	0.50	23	2.77	0.53	23	2.76	0.66	23	2.74	0.54	22	2.79	0.51
MBSR	30	2.78	0.47	25	2.72	0.48	24	2.78	0.52	24	2.60	0.52	24	2.63	0.56
Good															
WL	30	3.96	0.60	28	3.91	0.60	28	3.91	0.66	28	3.89	0.58	26	3.98	0.67
HEMBP	29	3.96	0.45	23	4.07	0.52	23	4.05	0.48	23	3.94	0.49	22	3.99	0.45
MBSR	30	4.03	0.44	25	4.03	0.37	23	4.12	0.46	24	3.85	0.40	24	3.91	0.44
Safe															
WL	30	3.88	0.82	28	3.93	0.80	28	3.94	0.78	28	3.84	0.64	26	3.99	0.76
HEMBP	29	3.77	0.60	23	4.04	0.66	23	4.05	0.58	23	3.86	0.73	22	3.94	0.54
MBSR	30	3.91	0.49	25	4.11	0.29	23	4.24	0.42	24	3.80	0.48	24	3.95	0.49
Enticing															
WL	30	4.69	0.71	28	4.56	0.66	28	4.50	0.76	28	4.64	0.63	26	4.63	0.68
HEMBP	29	4.61	0.57	23	4.69	0.57	23	4.57	0.59	23	4.46	0.56	22	4.55	0.49
MBSR	30	4.78	0.51	25	4.72	0.49	23	4.77	0.53	24	4.64	0.48	24	4.63	0.52
Alive															
WL	30	3.57	0.75	28	3.45	0.72	28	3.50	0.85	28	3.51	0.82	26	3.57	0.94
HEMBP	29	3.73	0.64	23	3.72	0.80	23	3.75	0.68	23	3.70	0.63	22	3.68	0.75
MBSR	30	3.74	0.70	25	3.54	0.70	23	3.64	0.77	24	3.38	0.61	24	3.47	0.69
Fluid															
WL	30	3.65	0.42	28	3.68	0.48	28	3.65	0.47	28	3.72	0.39	26	3.73	0.41
HEMBP	29	3.70	0.60	23	3.56	0.55	23	3.51	0.52	23	3.57	0.68	22	3.51	0.60
MBSR	30	3.75	0.38	25	3.64	0.41	23	3.61	0.44	24	3.70	0.45	24	3.74	0.30
Empowering															
WL	30	4.13	0.63	28	4.26	0.59	28	4.20	0.71	28	4.18	0.62	26	4.25	0.58
HEMBP	29	4.30	0.59	23	4.07	0.68	23	4.04	0.63	23	4.00	0.65	22	4.12	0.59
MBSR	30	4.13	0.73	25	4.17	0.67	23	4.16	0.76	24	4.18	0.84	24	4.18	0.55
Communal															
WL	30	3.88	0.66	28	3.92	0.68	28	3.95	0.68	28	3.85	0.62	26	4.00	0.67
HEMBP	29	3.98	0.57	23	4.09	0.49	23	4.07	0.47	23	3.96	0.49	22	3.93	0.51
MBSR	30	3.93	0.50	25	4.06	0.49	23	4.13	0.56	24	3.89	0.55	24	3.87	0.54
Funny															
WL	30	3.87	0.89	28	3.88	0.95	28	3.96	0.96	28	4.13	0.88	26	4.12	0.86
HEMBP	29	4.13	0.85	23	4.32	0.61	23	4.17	0.76	23	4.14	0.67	22	4.12	0.77
MBSR	30	4.22	0.77	25	4.29	0.62	23	4.61	0.80	24	4.18	0.93	24	4.38	0.90

*WL*, wait-list control group; *HEMBP*, humor-enriched mindfulness-based program; *MBSR*, mindfulness-based stress reduction; *FU*, follow-up; *PWB*, psychological well-being

**Table 4:** Pearson correlations of outcome variables at pre-test

Outcome	Fun	Ben	Non	Wit	Iro	Sat	Sar	Cyn	(1)	(2)	(3)	(4)	(5)	(6)
(1) Mindfulness	0.11	0.47**	0.07	0.24*	− 0.08	0.10	− 0.17	− 0.12						
(2) PWB	0.13	0.32**	− 0.06	0.07	− 0.24*	− 0.11	− 0.18	− 0.32**	0.62**					
(3) Life satisfaction	0.17	0.22*	0.15	0.02	− 0.19	− 0.06	− 0.14	− 0.26*	0.40**	0.77**				
(4) Stress	− 0.14	− 0.31**	− 0.06	− 0.04	0.09	− 0.06	0.11	0.14	− 0.60**	− 0.68**	− 0.53**			
(5) Anxiety	− 0.08	− 0.15	− 0.10	0.05	0.22*	0.12	0.22*	0.18	− 0.55**	− 0.41**	− 0.25*	0.65**		
(6) Depression	− 0.24*	− 0.16	− 0.05	0.05	0.13	0.01	0.09	0.20	− 0.48**	− 0.69**	− 0.55**	0.63**	0.49**	
Good	0.14	0.24*	− 0.02	0.03	− 0.21*	− 0.17	− 0.28**	− 0.42**	0.40**	0.65**	0.52**	− 0.43**	− 0.22*	− 0.47**
Safe	0.17	0.39**	0.08	0.16	− 0.16	− 0.09	− 0.11	− 0.29**	0.42**	0.67**	0.57**	− 0.50**	− 0.29**	− 0.47**
Enticing	0.06	0.01	− 0.13	− 0.03	− 0.27**	− 0.21*	− 0.23*	− 0.40**	0.23*	0.58*	0.44**	− 0.39**	− 0.24*	− 0.45**
Alive	0.16	0.07	− 0.04	− 0.04	− 0.07	− 0.05	− 0.29**	− 0.30**	0.26*	0.38**	0.28**	− 0.18	0.01	− 0.28**
Fluid	0.00	− 0.02	0.00	0.09	0.21*	0.31**	0.16	0.28**	− 0.08	− 0.17	− 0.21*	0.19	0.29**	0.17
Empowering	0.21*	0.12	− 0.07	0.11	0.02	0.15	− 0.01	− 0.01	0.14	0.15	0.12	− 0.16	− 0.07	− 0.19
Communal	− 0.11	0.27*	0.14	− 0.02	− 0.29**	− 0.26*	− 0.41**	− 0.42**	0.23*	0.33**	0.27*	− 0.21	− 0.10	− 0.13
Funny	0.31**	0.44**	0.19	0.28**	0.03	0.10	0.02	− 0.10	0.44**	0.44**	0.36**	− 0.46**	− 0.21*	− 0.37**

*Ben*, benevolent humor; *Non*, nonsense; *Iro*, irony; *Sat*, satire; *Sar*, sarcasm; *Cyn*, cynicism; *PWB*, psychological well-being. *N* = 89

^*^p < 0.05; **p < 0.01

Furthermore, no significant differences based on completion status were detected at pre-test for all demographic variables (Table 1), as well as in the mean levels of mindfulness, fun, benevolent humor, nonsense, wit, irony, satire, sarcasm, cynicism, psychological well-being, perceived stress, symptoms of anxiety and depression, and primal world beliefs (Table 2). However, completers scored significantly higher in life satisfaction at pre-test than dropouts. Additionally, dropout rates showed no significant variation between groups, suggesting a lack of systematic dropout (*χ*^2^(2, 89) = 1.14, *p* = 0.57).

Participants in the HEMBP attended on average 6.83 classes (*SD* = 0.86) and in MBSR 6.88 classes (*SD* = 0.68). As displayed in Table 5, participants in the HEMBP reported an average daily formal mindfulness practice time of 13.87 min (*SD* = 6.59) during the training period, whereas participants in MBSR reported 21.48 min (*SD* = 12.61), resulting in a significant group difference (*t*(36.84) = − 2.65, *p* = 0.01). However, the average daily formal practice time among participants who completed the follow-up measurements approached a similar level and no further significant group differences were found (all *p* ≥ 0.20).

**Table 5:** Daily formal mindfulness practice outside of class by group

Duration (min)	During training period		1-month follow-up		3-month follow-up		6-month follow-up	
	HEMBP	MBSR	HEMBP	MBSR	HEMBP	MBSR	HEMBP	MBSR
	*n* = 23	*n* = 25	*n* = 23	*n* = 24	*n* = 23	*n* = 24	*n* = 22	*n* = 24
0	0 (0.00%)	0 (0.00%)	3 (13.04%)	8 (33.33%)	10 (43.48%)	13 (54.17%)	9 (40.91%)	14 (58.33%)
1–10	11 (47.83%)	8 (32.00%)	16 (69.57%)	8 (33.33%)	12 (52.17%)	7 (29.17%)	12 (54.55%)	3 (12.50%)
11–20	9 (39.13%)	7 (28.00%)	4 (17.39%)	6 (25.00%)	1 (4.35%)	2 (8.33%)	1 (4.55%)	7 (29.17%)
> 20	3 (13.04%)	10 (40.00%)	0 (0.00%)	2 (8.33%)	0 (0.00%)	2 (8.33%)	0 (0.00%)	0 (0.00%)
*M* (*SD*)	13.87 (6.59)	21.48 (12.61)	7.35 (5.78)	8.04 (9.54)	3.61 (4.25)	6.67 (10.50)	3.68 (4.87)	5.54 (7.58)

*HEMBP*, humor-enriched mindfulness-based program; *MBSR*, mindfulness-based stress reduction

### Program Effectiveness

Results of the linear mixed-effects models are presented in Table 6. Participants in the wait-list control did not exhibit significant changes in outcome measures over time. Compared to the wait-list control group, the models predicted a significant increase in mindfulness for participants in both programs from pre- to post-test (HEMBP: *b* = 0.12, *p* = 0.01; MBSR: *b* = 0.15, *p* = 0.001) and those effects remained unchanged during the follow-up period (HEMBP: *b* = − 0.01, *p* = 0.56; MBSR: *b* = − 0.01, *p* = 0.43). Compared to the wait-list control group, the models predicted greater increases over time (a) in life satisfaction for both participants in the HEMBP (*b* = 0.06, *p* = 0.02) and MBSR (*b* = 0.06, *p* = 0.01), and (b) in psychological well-being (*b* = 0.03, *p* = 0.04) and in benevolent humor (*b* = 0.05, *p* = 0.03) for HEMBP participants. Compared to the wait-list control group, the models predicted significant greater decreases over time in perceived stress (*b* = − 0.04, *p* < 0.05) and the primary primal *good* (*b* = − 0.03, *p* = 0.01) for MBSR.

**Table 6:** Results from linear mixed-effects model analyses of the outcome variables

Outcome	Intercept			Slope (Time)		
	*b*	*p*	95% CI	*b*	*p*	95% CI
Mindfulness				Time 1		
WL	3.54	< 0.001	[3.35; 3.74]	0.05	0.15	[− 0.02; 0.11]
HEMBP	0.14	0.33	[− 0.14; 0.43]	0.12	0.01	[0.03; 0.21]
MBSR	0.11	0.46	[− 0.18; 0.39]	0.15	0.001	[0.06; 0.24]
HEMBPvsMBSR				0.03	0.50	[− 0.06; 0.13]
				Time 2		
WL				<0.01	0.84	[–0.02; 0.02]
HEMBP				–0.01	0.56	[–0.04; 0.02]
MBSR				–0.01	0.43	[–0.05; 0.02]
HEMBPvsMBSR				<0.01	0.85	[–0.04; 0.03]
Fun						
WL	4.31	< 0.001	[3.96; 4.65]	0.02	0.13	[− 0.01; 0.06]
HEMBP	< 0.01	0.99	[− 0.50; 0.51]	− 0.03	0.19	[− 0.08; 0.02]
MBSR	− 0.09	0.73	[− 0.59; 0.41]	− 0.03	0.23	[− 0.07; 0.02]
HEMBPvsMBSR				< 0.01	0.90	[− 0.04; 0.05]
Humor						
WL	4.70	< 0.001	[4.42; 4.98]	− 0.02	0.21	[− 0.05; 0.01]
HEMBP	0.05	0.81	[− 0.35; 0.45]	0.05	0.03	[0.01; 0.09]
MBSR	0.31	0.13	[− 0.09; 0.71]	0.01	0.62	[− 0.03; 0.05]
HEMBPvsMBSR				− 0.04	0.09	[− 0.08; 0.01]
Nonsense						
WL	4.62	< 0.001	[4.19; 5.04]	− 0.03	0.12	[− 0.07; 0.01]
HEMBP	0.31	0.32	[− 0.31; 0.93]	0.03	0.36	[− 0.03; 0.08]
MBSR	− 0.16	0.61	[− 0.77; 0.46]	0.02	0.50	[− 0.04; 0.07]
HEMBPvsMBSR				− 0.01	0.80	[− 0.06; 0.05]
Wit						
WL	4.34	< 0.001	[3.96; 4.72]	< 0.01	0.90	[− 0.03; 0.03]
HEMBP	0.13	0.65	[− 0.42; 0.68]	0.02	0.35	[− 0.02; 0.06]
MBSR	− 0.09	0.75	[− 0.64; 0.46]	0.03	0.19	[− 0.01; 0.07]
HEMBPvsMBSR				0.01	0.73	[− 0.04; 0.05]
Irony						
WL	3.89	< 0.001	[3.53; 4.24]	0.01	0.73	[− 0.03; 0.04]
HEMBP	0.06	0.83	[− 0.46; 0.57]	− 0.04	0.09	[− 0.09; 0.01]
MBSR	0.20	0.43	[− 0.30; 0.71]	− 0.02	0.32	[− 0.07; 0.02]
HEMBPvsMBSR				0.02	0.46	[− 0.03; 0.07]
Satire						
WL	3.71	< 0.001	[3.35; 4.08]	− 0.01	0.45	[− 0.05; 0.02]
HEMBP	0.18	0.49	[− 0.35; 0.71]	0.03	0.26	[− 0.02; 0.09]
MBSR	0.23	0.39	[− 0.29; 0.75]	0.04	0.12	[− 0.01; 0.10]
HEMBPvsMBSR				0.01	0.70	[− 0.05; 0.07]
Sarcasm						
WL	3.31	< 0.001	[2.89; 3.73]	− 0.01	0.65	[− 0.05; 0.03]
HEMBP	0.06	0.85	[− 0.56; 0.67]	0.02	0.58	[− 0.04; 0.07]
MBSR	0.07	0.81	[− 0.53; 0.68]	0.03	0.22	[− 0.02; 0.09]
HEMBPvsMBSR				0.02	0.53	[− 0.04: 0.08]
Cynicism						
WL	3.37	< 0.001	[2.98; 3.75]	− 0.01	0.89	[− 0.04; 0.04]
HEMBP	− 0.26	0.36	[− 0.82; 0.30]	0.02	0.42	[− 0.04; 0.08]
MBSR	0.13	0.65	[− 0.42; 0.68]	0.03	0.34	[− 0.03; 0.09]
HEMBPvsMBSR				< 0.01	0.90	[− 0.06; 0.07]
PWB						
WL	5.18	< 0.001	[4.93; 5.44]	− 0.01	0.39	[− 0.03; 0.01]
HEMBP	− 0.09	0.65	[− 0.46; 0.28]	0.03	0.04	[0.01; 0.07]
MBSR	0.10	0.59	[− 0.27; 0.47]	0.03	0.08	[0.00; 0.06]
HEMBPvsMBSR				− 0.01	0.73	[− 0.04; 0.03]
Life satisfaction						
WL	4.81	< 0.001	[4.43; 5.19]	− 0.02	0.17	[− 0.06; 0.01]
HEMBP	− 0.20	0.47	[− 0.75; 0.35]	0.06	0.02	[0.01; 0.11]
MBSR	0.02	0.95	[− 0.53; 0.56]	0.06	0.01	[0.01; 0.11]
HEMBPvsMBSR				< 0.01	0.90	[− 0.05; 0.05]
Stress						
WL	2.83	< 0.001	[2.65; 3.00]	0.01	0.26	[− 0.01; 0.04]
HEMBP	0.03	0.81	[− 0.22; 0.29]	− 0.02	0.21	[− 0.06; 0.01]
MBSR	− 0.06	0.67	[− 0.31; 0.20]	− 0.04	< 0.05	[− 0.07; − 0.01]
HEMBPvsMBSR				− 0.01	0.48	[− 0.05; 0.02]
Good						
WL	3.93	< 0.001	[3.75; 4.11]	< 0.01	0.66	[− 0.01; 0.02]
HEMBP	0.08	0.54	[− 0.18; 0.34]	− 0.01	0.37	[− 0.03; 0.01]
MBSR	0.11	0.39	[− 0.14; 0.36]	− 0.03	0.01	[− 0.05; − 0.01]
HEMBPvsMBSR				− 0.02	0.08	[− 0.04; 0.00]
Safe						
WL	3.89	< .001	[3.67; 4.11]	0.01	0.37	[− 0.01; 0.03]
HEMBP	− 0.01	.93	[− 0.33; 0.30]	< 0.01	0.83	[− 0.03; 0.03]
MBSR	0.11	.49	[− 0.20; 0.42]	− 0.03	< 0.05	[− 0.06; − 0.01]
HEMBPvsMBSR				− 0.03	0.10	[− 0.06; 0.00]
Enticing						
WL	4.61	< 0.001	[4.41; 4.81]	< 0.01	0.61	[− 0.01; 0.02]
HEMBP	0.04	0.81	[− 0.26; 0.33]	− 0.02	0.11	[− 0.05; 0.00]
MBSR	0.16	0.28	[− 0.13; 0.45]	− 0.03	0.03	[− 0.05; − 0.01]
HEMBPvsMBSR				− 0.01	0.60	[− 0.03; 0.02]
Alive						
WL	3.52	< 0.001	[3.27; 3.77]	< 0.01	0.87	[− 0.02; 0.02]
HEMBP	0.21	0.26	[− 0.16; 0.57]	− 0.01	0.44	[− 0.04; 0.02]
MBSR	0.16	0.39	[− 0.20; 0.52]	− 0.03	0.02	[− 0.06; − 0.01]
HEMBPvsMBSR				− 0.02	0.15	[− 0.05; 0.01]
Fluid						
WL	3.64	< 0.001	[3.48; 3.80]	0.01	0.20	[− 0.01; 0.03]
HEMBP	< 0.01	0.99	[− 0.24; 0.24]	− 0.03	0.03	[− 0.05; − 0.01]
MBSR	0.06	0.62	[− 0.18; 0.29]	< 0.01	0.79	[− 0.03; 0.02]
HEMBPvsMBSR				0.02	0.06	[0.00; 0.05]
Empowering						
WL	4.17	< 0.001	[3.96; 4.39]	0.01	0.24	[− 0.01; 0.04]
HEMBP	0.02	0.90	[− 0.29; 0.33]	− 0.03	0.06	[− 0.07; 0.00]
MBSR	− 0.05	0.75	[− 0.36; 0.26]	− 0.01	0.53	[− 0.05; 0.02]
HEMBPvsMBSR				0.02	0.21	[− 0.01; 0.06]
Communal						
WL	3.89	< 0.001	[3.70; 4.07]	0.01	0.37	[− 0.01; 0.03]
HEMBP	0.17	0.22	[− 0.10; 0.44]	− 0.02	0.13	[− 0.05; 0.01]
MBSR	0.13	0.34	[− 0.14; 0.40]	− 0.03	< 0.05	[− 0.06; − 0.01]
HEMBPvsMBSR				− 0.01	0.67	[− 0.04; 0.02]
Funny						
WL	3.86	< 0.001	[3.59; 4.14]	0.04	0.01	[0.01; 0.08]
HEMBP	0.30	0.13	[− 0.10; 0.70]	− 0.06	0.01	[− 0.11; − 0.01]
MBSR	0.39	0.05	[0.00; 0.79]	− 0.05	0.06	[− 0.09; 0.00]
HEMBPvsMBSR				0.01	0.55	[− 0.03; 0.06]

*WL*, wait-list control group; *HEMBP*, humor-enriched mindfulness-based program; *MBSR*, mindfulness-based stress reduction; *PWB*, psychological well-being. The estimates (*b*’s) for the intercept for the training groups (HEMBP, MBSR) indicate whether they significantly differ from the wait-list control group (WL) at pre-test. The estimates (*b*’s) for the slopes indicate (1) whether there is a significant change in the respective outcome variable over time in the wait-list group (WL); (2) whether the change in the wait-list group significantly differs from that observed in the respective training group (HEMBP, MBSR = training effects), with positive values indicating an increase, negative values indicating a reduction; and (3) whether there is a significant difference between the training groups (HEMBPvsMBSR) regarding training effects, with positive values indicating that the training effect for MBSR is larger than for the HEMBP, and negative values indicating that the training effect for the HEMBP is larger than in MBSR. Time 1 (training period) and Time 2 (follow-up period) refer to the two different time phases for the piecewise-growth model

There were no significant differences in the rate of change over time between the training groups (HEMBPvsMBSR). However, there was a trend toward a greater increase in benevolent humor (*b* = − 0.04, *p* = 0.09) and toward a greater decrease in *good* (*b* = − 0.02, *p* = 0.08) for MBSR compared to the HEMBP.

### Exploratory Analyses

Exploratory analyses were conducted for the secondary primals *safe*, *enticing*, *alive*, *fluid*, *empowering*, and *communal*, as well as the tertiary primal *funny*. No significant changes in these outcome variables were observed for participants in the wait-list control group over time, except for a significant increase in the primal *funny* (*b* = 0.04, *p* = 0.01). Compared to the wait-list control group, the models predicted significant greater decreases over time in *fluid* (*b* = − 0.03, *p* = 0.03) and *funny* (*b* = − 0.06, *p* = 0.01) for the HEMBP, as well as in *safe* (*b* = − 0.03, *p* < 0.05), *enticing* (*b* = − 0.03, *p* = 0.03), *alive* (*b* = − 0.03, *p* = 0.02), and *communal* (*b* = − 0.03, *p* < 0.05) for MBSR. As the wait-list control group exhibited a significant increase in *funny* (*b* = 0.06, *p* = 0.01), it was explored whether there existed a potential within-group effect exclusive to the HEMBP, which did not reach statistical significance (*b* = − 0.02, *p* = 0.37). Thus, the observed decrease in *funny* associated with the HEMBP compared to the wait-list (*b* = 0.04, *p* = 0.01) is attributed to the observed increase in *funny* in the wait-list control group.

There were no significant differences in the rate of change over time between the training groups (HEMBPvsMBSR). However, there was a trend toward a greater decrease in *fluid* (*b* = 0.02, *p* = 0.06) for the HEMBP compared to MBSR and toward a greater decrease in *safe* (*b* = − 0.03, *p* = 0.10) for MBSR compared to the HEMBP.

Exploratory analysis of dose–response effects of practice time showed that average practice time during the training period was significantly correlated with change scores from pre- to post-test for fun (HEMBP: *r* = 0.46, *p* = 0.03) and satire (MBSR: *r* = 0.48, *p* = 0.02), and marginally significantly for mindfulness (HEMBP: *r* = 0.37, *p* = 0.08; MBSR: *r* = 0.38, *p* = 0.06) and fun (MBSR: *r* = 0.39, *p* = 0.06). There were also significant associations between average practice time during the follow-up period and change scores from post-test to 6-month follow-up for mindfulness (HEMBP: *r* = 0.51, *p* = 0.02) and benevolent humor (HEMBP: *r* = 0.73, *p* < 0.001), and marginally significantly for cynicism (MBSR: *r* = − 0.39, *p* = 0.06) and life satisfaction (HEMBP: *r* = 0.37, *p* = 0.09).

## Discussion

Results of this randomized controlled trial provide evidence for the efficacy of the HEMBP in increasing mindfulness, benevolent humor, psychological well-being, and life satisfaction compared to the wait-list control group. MBSR was found to be effective in increasing mindfulness and life satisfaction, and for decreasing stress and the primal world belief *good* compared to the wait-list control group. Exploratory analyses of secondary primal world beliefs and *funny* revealed that individuals in the HEMBP showed decreases in *fluid* and *funny*, and participants in MBSR showed a decrease in *safe*, *enticing*, *alive*, and *communal* compared to the wait-list control group. However, there was also an increase of *funny* in the wait-list condition. No significant differences between the training groups were found, except for a trend toward a greater increase in benevolent humor in the HEMBP than MBSR, and toward a greater decrease in *good* and *safe* (MBSR), and in *fluid* (the HEMBP).

This study largely replicates previous HEMBP findings (Kastner, 2024). One difference pertains to its effects on humor. Although participation in the HEMBP led to increases in mindfulness and benevolent humor, indicative of the development of a mindful-humorous perspective, no decreases in sarcasm and cynicism were observed. This discrepancy likely stems from variations in study advertising. In the current study emphasis on humor in advertisements was minimized to mitigate potential expectancies among participants primarily interested in humor or a combined approach when assigned to a “mindfulness-only” training (i.e., MBSR). Hence, the programs were primarily promoted as mindfulness-based, with minimal mention of humor (i.e., “humor *might* be part of some trainings”). Feedback and evaluations indicated that this approach elicited disappointed expectations in the HEMBP, with some participants anticipating a mindfulness-only training. There were prior expectations for a greater emphasis on mindfulness, and as a consequence, some participants expressed reluctance to engage in humor-related discussions and exercises during classes. However, as noted by Kastner (2024), inhibiting or reducing potentially destructive forms of humor requires self-observation and reflection on its potential negative consequences on oneself and others. In contrast, conveying the benefits of a mindful-humorous perspective was easier, explaining the increase in benevolent humor specifically for the HEMBP despite these influences, while no significant changes in the other humor styles were observed.

Conversely, no changes in participants’ habitual use of benevolent humor, sarcasm, and cynicism were observed in MBSR, contrary to hypotheses and predictions of the mindful humor filter model. This draws attention to ethical considerations associated with mindfulness practice. In the HEMBP, discussions about the distinction between laughing with and laughing at others explicitly include an ethical dimension. In contrast, ethics in MBSR are not explicitly addressed or taught, but are rather assumed to be embodied by the MBSR teacher and often integrated into the ethical code of conduct of the institutional framework, such as the Hippocratic oath in clinical settings (Kabat-Zinn, 2011). However, criticisms have been raised that this might not adequately serve as an ethical basis for MBPs (e.g., Shonin et al., 2015; Stanley et al., 2018). Although this study was not designed to test such contentions, the results suggest that an 8-week training period may be too short for mindfulness alone to substantially influence intentions regarding the use of less harmful or destructive forms of humor. This highlights the importance of long-term practice. However, humor as a character strength, representing a positively valued, virtuous trait (Peterson & Seligman, 2004), improved after participation in a mindfulness-based strengths program (MBSP; Niemiec, 2014) and MBSR (Hofmann et al., 2020), indicating the need for additional research.

Regarding the efficacy of the trainings in promoting well-being-related outcomes, no group differences were observed between the HEMBP and MBSR. However, only participation in the HEMBP was associated with an increase in psychological well-being, whereas MBSR led to a decrease in stress. This divergence may be a result of MBSR’s emphasis on stress reduction in contrast to the HEMBP’s focus on enhancing positive qualities, such as, a mindful-humorous perspective, mindful humor, loving-kindness, and gratitude. However, it is worth noting that the HEMBP has also demonstrated stress reduction (Kastner, 2024), warranting further research to validate these findings.

Moreover, both trainings showed similar effects in increasing mindfulness, which persisted for up to 6 months after completion. Attrition rates did not differ between groups, and attendance in the training sessions was consistently high. Throughout the training period, participants in MBSR reported longer average daily formal practice time compared to those in the HEMBP, likely due to shorter guided meditations in the latter. However, this difference evened out at the first follow-up. In addition, there was a tendency toward more participants in MBSR not continuing formal mindfulness practice during the follow-up period than in the HEMBP (1-month: 33.33% MBSR vs. 13.04% HEMBP; 3-month: 54.16% vs. 43.48%; 6-month: 58.33% vs. 40.91%). This aligns with the HEMBP’s principle that ongoing mindfulness practice is vital for integrating mindfulness into daily life, akin to MBSR. Nonetheless, the HEMBP also encourages participants to practice, even if for shorter durations, emphasizing the importance of continuity over complete cessation of practice. Longer formal practice during the training period went along with a trend toward higher increases in mindfulness in both groups. During the follow-up period, continued mindfulness practice strongly correlated with increases in mindfulness and benevolent humor only in the HEMBP, suggesting that sustained mindfulness practice seems to be especially relevant for cultivating a mindful-humorous perspective.

The study also examined whether participation in a MBP could influence individuals’ primal world beliefs. While mindfulness was positively correlated with the primal world beliefs *good*, *safe*, *enticing*, and *alive*, inducing changes in these primals presented a complex picture. Contrary to expectations, no change in the general factor *good* was observed among HEMBP participants, while participation in MBSR was even associated with a reduction. Exploratory analyses of the secondary primals were conducted to elucidate potential factors explaining these findings. For the HEMBP, decreases were noted in *fluid* and *funny*. The notion of fluidity includes the belief that things are fragile and easily breakable, which should diminish when adopting the perspective that life is imperfect, implying that things are bound to break, after all. Still, a decrease in *fluid* coincides with perceiving the world as more solid, and less in flux. This somehow contradicts the mindful-humorous perspective, which embraces the ever-changing nature of all experiences. Yet, an experiential understanding of impermanence likely requires continuous long-term mindfulness practice rather than a purely cognitive comprehension that objects or situations are subject to change. The observed decrease in *funny* should be interpreted cautiously, as it may be attributed to the increase observed in the wait-list control condition. This increase was of a similar magnitude to the observed decrease in both training groups. Accordingly, explorative analysis of a potential within-group effect in the HEMBP revealed no time effect.

Results for MBSR revealed a distinct pattern. The drivers behind the overall decrease in *good* can be attributed to the reductions in *safe*, *enticing*, *alive*, and *communal*. This shift may initially stem from the focus of MBSR on stress reduction and the realization that unpleasant experiences are an inherent part of life. MBSR was developed to alleviate suffering among chronic pain patients, focusing on coping with unpleasant experiences of pain and stress through acceptance (Kabat-Zinn, 1990, 2011). This emphasis may have contributed to the perception of the world as less safe, enticing, and alive, and more hierarchical (i.e., less communal), potentially reflecting an initial tendency toward a more negative outlook. However, the assumption is that such a change or initial decline is likely temporary. The expectation is that mindfulness practice would eventually lead to an increase in *good*, although this change might largely depend on consistent, sustained practice. In general, there was a trend for MBSR to be associated with greater decreases in *good* and *safe* than the HEMBP, which could reflect the HEMBPs focus on nourishing a mindful-humorous perspective on life.

### Limitations and Future Directions

Methodological strengths of this study were the employment of a randomized controlled trial and the use of advanced data analysis methods to probe different approaches for integrating time into the models, facilitating the detection of non-linear growth in the data, such as in mindfulness. The study has also several limitations. The interpretation of the effects of the HEMBP on humor may be affected by the limited mention of humor in the study advertisements. Future studies comparing the HEMBP with mindfulness-only trainings should explicitly emphasize the role of humor as an important component of certain training groups and proactively communicate the research interest in the intersection of mindfulness and humor, as well as in potential effects of MBPs on humor, to better manage expectancy effects. However, disappointed expectations were limited to a few participants in the HEMBP, with the majority actively participating in regular mindfulness practice, group discussions, and exercises, as reflected in the observed effects on outcome variables. Second, generalizability is limited because of convenience sampling. However, efforts were made to address this by broadly distributing advertisements to recruit a diverse participant sample, as evidenced by the demographics, which were balanced between groups. Moreover, an important question is whether the focus should be on generalizing to a truly random sample or to the population of individuals potentially interested in participating in a MBP or the HEMBP (Rosenkranz et al., 2019). Still, future studies should aim to replicate these findings with more representative and also non-WEIRD samples. Third, the current study exclusively relied on self-reports, potentially resulting in common method variance (Podsakoff et al., 2003). This could be mitigated by incorporating additional data sources or employing objective measures, such as observational or psychophysiological.

Now that the efficacy of the HEMBP has been quantitatively demonstrated across two randomized controlled trials, it is imperative to explore qualitative questions. Joseph Goldstein hints at the potential for a mindful-humorous perspective to naturally emerge through continuous introspection into one’s mind:

Another quality that develops in meditation is a sense of humor about our minds, our lives, and the human predicament. If you do not have a sense of humor now, meditate for a while and it will come, because it’s difficult to watch the mind steadily and systematically without learning to smile. (Goldstein, 2011, p. 25)

The HEMBP has demonstrated its capacity to foster such a perspective. However, the extent to which a mindful-humorous outlook enhances mindfulness practice, such as in cultivating a novel relationship to hindrances during meditation, ultimately befriending all experiences, and how such a process can be further facilitated and deepened, remains an open question. Future studies could use diary methodologies, systematically documenting participants’ experiences throughout the 8-week HEMBP. Further, semi-structured interviews could be conducted to explore moderators that promote or impede the adoption of a mindful-humorous perspective. Another line of research should investigate the intricate relationship between mindfulness and primal world beliefs in more detail, including the long-term potential of MBPs to impact such beliefs.

A practical concern lies in the dissemination of the HEMBP and its independent evaluation, which would contribute to additional support for its efficacy. Furthermore, investigating the applicability of the HEMBP in clinical settings for specific diagnoses or transdiagnostic purposes, and determining the conditions under which it is most effective, appears equally critical (Kastner, 2024). Moreover, future research endeavors should assess whether abbreviated versions, such as a 4-week program, yield comparable effects to the full 8-week program. The HEMBP, designed as a full-fledged MBP, enriches the expanding array of mindfulness-based programs by presenting a more lighthearted approach to mindfulness practice. This approach aims to encourage the initiation and sustained engagement in long-term mindfulness practice, offering an alternative pathway that complements, yet differs from, the more intense and attention-focused 45-min formal meditation requirements of programs like MBSR or Mindfulness-Based Cognitive Therapy (MBCT; Segal et al., 2013).

Finally, “lighthearted” should not be equated or conflated with “easy.” Quite the opposite, life will remain as challenging or effortless as before. Nevertheless, a mindful-humorous perspective has the potential to profoundly alter one’s relationship with life’s experiences as they are, regardless of whether they are hard or easy, pleasant or unpleasant. Some individuals may naturally possess a sense of lightheartedness and may not feel the need to participate in a HEMBP or similar approach. However, for those with a strong goal-oriented mindset, particularly in individualized Western cultures, adopting a lighthearted approach might be precisely what is needed: achieving goals through letting go.

In summary, the findings of this study closely correspond with existing evidence regarding the validity and efficacy of the HEMBP in enhancing mindfulness, benevolent humor, psychological well-being, and life satisfaction, proving as effective as the well-established MBSR. Both the HEMBP and MBSR demonstrated similar effects on outcomes. However, only participation in the HEMBP was associated with an increase in benevolent humor and psychological well-being, while participation in MBSR was associated with stress reduction. The study replicates findings that benevolent humor, which holds significance for both intra- and interpersonal well-being can be modified and enhanced alongside mindfulness. No effects on the primal *good* were observed for the HEMBP, while participation in MBSR was even associated with decreases in several primals. These short-term changes may reflect transient effects rather than enduring shifts. It would be premature to conclude that mindfulness alone cannot initiate positive changes in one’s sense of humor or worldviews. Instead, substantial changes aligning with the mindful humor filter model may require a long-term perspective contingent upon continuous mindfulness practice and discernment. The integration of an explicit ethical dimension into MBPs may reinforce this process. Further research is needed to investigate qualitative aspects of the integration of humor in MBPs and the long-term impact of MBPs on individuals’ worldviews. The unique features of the HEMBP in nourishing a mindful-humorous perspective and fostering mindful humor could allow for the exploration of new populations for MBPs, thereby expanding their reach. The HEMBP may serve as an intermediary approach by integrating an explicit ethical dimension addressing both light and darker humor qualities in an inclusive manner, thereby ensuring accessibility.

## Data Availability

The data described in this article are openly available on the Open Science Framework at: 10.17605/OSF.IO/D8WRN.

## Declarations

### Use of Artificial Intelligence

AI was used for editing the manuscript to improve English language.
